# Effect of the alcohol consumption on osteocyte cell processes: a molecular imaging study

**DOI:** 10.1111/jcmm.12113

**Published:** 2013-08-15

**Authors:** Delphine B Maurel, Delphine Benaitreau, Christelle Jaffré, Hechmi Toumi, Hugues Portier, Rustem Uzbekov, Chantal Pichon, Claude L Benhamou, Eric Lespessailles, Stéphane Pallu

**Affiliations:** a Laboratory of Oral Biology, School of DentistryKansas City, MO, USA; b Laboratory EA4708 I3MTO, Hôpital Porte Madeleine, Université d'OrléansOrléans Cedex, France; c Laboratoire M2S, UFR APS, Université de RennesRennes Cedex, France; d UFR STAPS, Université d'OrléansOrléans Cedex, France; e Département des Microscopies, Faculté de Médecine, Programme Pluriformation Analyse des Systèmes Biologiques, Université de ToursTours, France; f Centre de Biophysique Moléculaire, CBM, CNRS, Université d'OrléansOrléans Cedex, France

**Keywords:** osteocyte, alcohol, dose, lipid, lacunae, apoptosis

## Abstract

We have previously shown microarchitectural tissue changes with cellular modifications in osteocytes following high chronic alcohol dose. The aim of this study was to assess the dose effect of alcohol consumption on the cytoskeleton activity, the cellular lipid content and modulation of differentiation and apoptosis in osteocyte. Male Wistar rats were divided into three groups: Control (C), Alcohol 25% v/v (A25) or Alcohol 35% v/v (A35) for 17 weeks. Bone mineral density (BMD) was assessed by DXA, osteocyte empty lacunae, lacunae surface, bone marrow fat with bright field microscopy. Osteocyte lipid content was analysed with transmission electron microscopy (TEM) and epifluorescence microscopy. Osteocyte apoptosis was analysed with immunolabelling and TEM. Osteocyte differentiation and cytoskeleton activity were analysed with immunolabelling and real time quantitative PCR. At the end of the protocol, BMD was lower in A25 and A35 compared with C, while the bone marrow lipid content was increased in these groups. More empty osteocyte lacunae and osteocyte containing lipid droplets in A35 were found compared with C and A25. Cleaved caspase-3 staining and chromatin condensation were increased in A25 and A35 *versus* C. Cleaved caspase-3 was increased in A35 *versus* A25. CD44 and phosphopaxillin stainings were higher in A35 compared with C and A25. Paxillin mRNA expression was higher in A35 *versus* A25 and C and sclerostin mRNA expression was higher in A35 *versus* C. We only observed a dose effect of alcohol consumption on cleaved caspase-3 osteocyte immunostaining levels and on the number of lipid droplets in the bone marrow.

## Introduction

Chronic heavy alcohol consumption is one of the major causes of osteoporosis in men [1]. It has been shown that chronic heavy alcohol consumption changes the activity and number of osteoblasts and osteoclasts [2–4] and affects the thickness of the trabecular and cortical bone [5], leading to lower bone mineral density (BMD) [6,7] and higher risk of fractures [8].

There is a dose effect of chronic heavy alcohol consumption on parameters of bone macro- and microarchitecture. Our team has previously reported that the higher the consumption of alcohol is, the lower is the BMD, the cortical thickness and the higher is the pore number [9]. This study was performed with 2-month-old male Wistar rats receiving 29%, 34% or 38% of the kilocalories as alcohol [9]. A dose effect has also been reported on bone strength and bone repair, in 3-month-old male Wistar rats [10]. Ethanol decreased more bone strength and bone repair when it corresponded to 36% of total energy intake as compared to 26% [10].

It is actually well known that the macro- and microarchitecture are the results of bone remodelling. Bone formation and resorption are increased or decreased to adapt bone mass to its environment [11]. The osteocyte, the most abundant cell in bone, can control osteoblast and osteoclast activities, thereby controlling and modifying the balance between bone formation and resorption [12,13]. Our previous results have shown that a high alcohol regimen (35% ethanol v/v) induced 10-fold more osteocytes stained with cleaved caspase-3, and twofold more osteocytes stained with CD44 than control samples in cortical bone [14].

Our objective was to investigate if the macro- and microarchitectural tissue changes following alcohol consumption could be associated with cellular changes in the osteocytes of the cortical bone relative to the alcohol dose.

This cellular and molecular imaging study was designed to assess if the dose effect of alcohol consumption may affect the cytoskeleton activity, the cellular lipid content and modulate the differentiation and apoptosis of the osteocyte.

## Materials and methods

### Animals

Thirty-six male Wistar rats (Elevage Janvier, Le Genet-St-Isle, France) were acclimatized for 2 weeks and maintained under constant temperature (21 ± 2°C) and under 12-h/12-h light–dark cycles all along the experiment. The rats were housed two per standard cage and provided with a standard diet (M20, SDS, Argenteuil, France).

### Alcohol treatment

The rats were 8 weeks old at baseline. They were not skeletally mature; rats skeletal maturity occurs late (7 months old) compared with their sexual maturity (2 months old) [15]. We wanted to apply our model of alcohol treatment in the young adult. At 10 weeks of age, rats were randomly assigned to one of the following groups (*n* = 12 each): C: control rats, A25: rats fed ethanol at 7.3 g of ethanol/kg per day (25% v/v) or A35: rats fed 10.7 g of ethanol/kg of bodyweight/day (35% v/v). The alcohol treatment lasted 17 weeks. Therefore, including the 2 weeks of acclimation, rats were 27 weeks old at the end of the protocol. Rats from A25 and A35 groups drank *ad libitum* a solution composed of ethanol and water for 17 weeks. At the beginning of the protocol, the percentage of ethanol in the solution was 8% v/v and then it was progressively increased by adding 3% v/v every 3 days during the first 3 weeks to reach the final concentration of 25% v/v or 35% v/v. Food and beverage were separated to better mimic the human drinking pattern. The quantification of food eaten and beverage drank has been averaged for the two animals per cage. The quantity of food eaten and beverage drunk was controlled weekly in the alcohol-treated groups and the total amount of calorie intake was matched in the control group having only the solid food as a caloric supply. To perform the *in vivo* analysis, rats were anaesthetized with pentobarbital (Ceva Santé Animale, Libourne, France) diluted in sodium chloride (0.5 ml v/v). The procedure for the care and killing of the animals was in accordance with the European Community standards on the care and use of laboratory animals. The study was approved by a board institution and an ethics committee (agreement no. C45-234-9 and 2011-11-2) from the French Institute INSERM (Institut National de la Santé et de la Recherche Médicale) and from the agriculture council (Ministère de l'Agriculture, France, approval ID: INSERM45-001).

At the end of the study, the rats were anaesthetized with pentobarbital sodium (0.1 ml per 100 g of bw) and killed by cardiac exsanguination. Blood samples were collected and tibias were dissected free of connective and fat tissues. Left tibias were fixed in a 4% v/v formalin solution and right tibias were conserved at −80°C in RNA later (Qiagen, Qiagen Europe, Venlo, the Netherlands).

### Whole body fat mass and lean mass

At 10 and 27 weeks of age (before alcohol treatment and after the 17 weeks of alcohol treatment), bodyweight, lean and fat masses were measured by DXA (Discovery, Hologic, Bedford, MA, USA) using a specific small animal body composition mode calibrated with defined small animal phantom. This apparatus is commonly used in small animals and has been validated in our laboratory. The root-mean square C *versus* measurements where those published in Lespessailles *et al*. [16].

### Bone mineral content and BMD measurements

*In vivo* bone mineral content (BMC) and BMD of the left femur and whole body were measured at 10 and 27 weeks of age by DXA with a Discovery Hologic apparatus adapted to small animals. The root-mean square CV of *in vivo* whole body BMC and whole body BMD were, respectively, 1.2% and 0.87% and were determined from two repeated measurements with repositioning on thirty animals [16].

### Epifluorescence microscopy – immunostaining

Bone explants for epifluorescence microscopy were fixed in formalin 4% (v/v) for 4 days at +4°C. After 4 days, bone slices (thickness 400 μm) were cut transversally in the superior part of the tibia diaphysis with a high-speed rotary tool (Dremel 300; Dremel Europe, Breda, the Netherlands) equipped with a diamond saw, without previous embedding. Bone slices were decalcified with EDTA 177 g/l, pH 7.0–7.3 (Osteosoft, Merck KGaA, Darmstadt, Germany) for 24 hrs and then immunostained for epifluorescence microscopy. We have achieved two series of double immunostaining for epifluorescence. The first series was achieved with cleaved caspase-3 (Rabbit mAb Asp 175, Cell Signaling Technology Inc, Danvers, MA, USA), which is an early marker of apoptosis, at a dilution of 1:400, and CD44 (Santa Cruz Biotechnology, Santa Cruz, CA, USA) as a differentiation marker of osteocytes at a dilution of 1:200. The second series of experiments were performed with CD44 (at the previous dilution) and phospho-paxillin (Cell Signaling Technology Inc) at a dilution of 1:200. The phospho-paxillin (Tyr118) antibody detects endogenous levels of paxillin only when phosphorylated at tyrosine 118, and does not cross-react with other tyrosine phosphorylated proteins. Location of the stain was visualized with the secondary antibodies Dylight™ 488 anti-mouse at a dilution of 1:1000 for CD44, and Dylight™ 549 anti-rabbit (Rockland Immunochemicals, Gilbertsville, PA, USA) at a dilution of 1:1000 for both cleaved caspase-3 and phospho-paxillin, as described by Vatsa *et al*. [17].

Epifluorescence images of osteocytes were obtained with a Motic AE21 video camera attached to a Microvision microscope. The objective magnification was 10×. Eighteen rats were analysed (*n* = 5 for C, *n* = 5 for A35, *n* = 8 for A25) for each series of immunostaining. Five regions of interest around the cortical bone were analysed for each sample. The mean size of one region of interest (ROI) was about 300,000 μm^2^ and the total cortical area analysed was 1.5 mm^2^. We acquired images of cleaved caspase-3 and CD44 in each ROI for the first series of experiments, and images of CD44 and phospho-paxillin in each ROI for the second series. Images were analysed with Image J software (version 1.38). The number of cells stained per mm^2^ in each ROI was counted and the ratio of stained cells with each immunolabelling out of the cells stained with DAPI was calculated.

### Epifluorescence microscopy – Nile red staining

Bone explants were fixed in formalin 4% (v/v) for 4 days at +4°C. After 4 days, bone slices (thickness 400 μm) were cut transversally in the tibia diaphysis with a high-speed rotary tool (Dremel 300; Dremel) equipped with a diamond saw, without previous embedding.

Two bone slices were stained with Nile red fluorescent hydrophobic dye, based on the published protocol by Fowler and Greenspan [18]. This Nile red dye can specifically detect neutral ubiquitous lipid deposits that are usually not seen with oil red O or other traditional fat stains [18]. A stock solution of Nile red (1 mg/ml) was prepared in acetone and protected from light at 4°C. A working Nile red solution was freshly made by the addition of 50 μl of the stock solution to 10 ml of 75% glycerol, followed by a brisk mixing. A drop of this diluted Nile red solution was added to the samples placed on concave slides and then the slides were covered with a glass coverslip. After 5 min., the slice was examined by fluorescence microscopy. The same slices were also imaged by confocal microscopy 2 days later.

The slices stained with Nile red were imaged with fluorescent microscopy with an adapted fluorescent filter (630/60 nm). The objective magnification was ×40. Twenty-three rats were analysed (*n* = 7 in C group, *n* = 8 in A25 group, *n* = 8 in A35 group). Five regions of interest around the cortical bone were chosen and imaged for analysis of Nile red staining. The mean size of each ROI was 30,000 μm^2^ and the total cortical surface analysed represented 0.15 mm^2^. The images were analysed with Image J software to determine the number of Nile red positive stained cells.

### Light bright field microscopy

Bone explants from 19 rats (*n* = 6 in C group, *n* = 8 in A25 group, *n* = 5 in A35 group) were fixed in formalin 4% (v/v) for 4 days and then placed in EDTA solution for 24 hrs. Samples were then washed in 0.2 M sodium cacodylate buffer and were consequentially post- fixed with glutaraldehyde (1%) and formaldehyde (4%) mixture in 0.2 M cacodylate buffer (48 hrs) and osmium tetroxide (2%) in 0.2 M sodium cacodylate buffer (1 hr). Tibia fragments were dehydrated in alcohol of increasing concentrations and embedded in Epon resin.

From these blocks of resin, semi-thin sections (thickness 1 μm) were cut with a Leica ultra-microtome (Leica, Wetzlar, Germany), stained with standard Toluidine blue solution as described by Trump *et al*. [19]. They were then washed for 20 sec. in distilled water, for 20 sec. in 96% ethanol and again for 5 sec. in distilled water. After drying on heating plate (60°C, 30 min.), sections were mounted in Epoxy resin and covered by coverslips. After polymerization of the resin (48 hrs at 60°C), the preparations were analysed by light microscopy with Axioplan (Zeiss, Oberkochen, Germany) microscope equipped with a 11.2. Color Mosaic camera. Images of the cortical bone were acquired at 40× objective, while images of the bone marrow were acquired at 20× objective.

In each rat, five different cortical ROI were analysed. The ROIs were chosen to obtain the best representation of the whole bone slice. The mean size of each ROI was about 70,000 μm^2^ and therefore the whole region analysed represented a surface of about 0.35 mm^2^, which corresponded to 5% of the cortical bone available [20]. The osteocyte lacunae areas were determined with the Image J freehand tool. The number of lacunae without a cell inside per mm^2^ was counted.

Seventeen rats were used to analyse the adiposity of the bone marrow (*n* = 4 in C group, *n* = 8 in A25 group, *n* = 5 in A35 group). For each rat, four different ROIs of the bone marrow were analysed with Image J software. Their surfaces were about 100,000 μm^2^ and the total region analysed represented 0.4 mm^2^. The number of lipid droplets inside the ROI was counted and the mean surface area of the lipid droplets was assessed in each ROI by drawing the contour line of each lipid droplet with the Image J freehand tool.

### Transmission electron microscopy

From the same resin blocks, ultra-thin sections (75 nm) were cut with a Leica ultra-microtome (Leica), and placed on EM grids coated with Formvar films. Sections were placed in 4% (w/v) uranyl acetate (water solution, 20 min.) and lead citrate by Reynolds (5 min.) [21]. The sections were then observed with a JEM 1011 electron microscope (Jeol, Tokyo, Japan) equipped with a Gatan digital camera driven by Digital Micrograph software (Gatan, Pleasanton, CA, USA). For the osteocyte chromatin condensation evaluation, 10.3 ± 5.8 cells were analysed for each rat in the A35 group, 15.3 ± 8.2 cells for each rat in the A25 group and 10.2 ± 7.1 cells for each rat in the C group. The mean percentage of osteocytes, which presented a nucleus with a chromatin condensation, was given per group.

### Confocal microscopy analysis

The goal of confocal microscopy analysis was to visualize the Nile red staining in osteocytes and lacunar network in three dimension space, to appreciate lipid infiltration in the network. Confocal images of osteocytes were obtained with Zeiss Laser Scanning Microscope LSM 510 Meta confocal system attached to a Zeiss inverted microscope (Axiovert 200M; Zeiss). A Zeiss 40× oil immersion objective lens was used with a numerical aperture of 1.3 and a working distance of 210 μm to acquire images. The tibia bone explants were excited by an argon/2 laser at 488 nm and a helium/neon laser at 543 nm, respectively, to visualize Nile red staining. The emission wavelengths were collected at 520/40 nm and 600/40 nm. Sequences of x-y optical slices, measuring 1.1 μm in thickness, were collected as z-stacks, separated by 0.68 μm on the z axis. A single image of maximum projection was obtained from the z-stacks with LSM Image Examiner software. A typical z-stack contained about 20 slices and the final dimensions of the ROI studied were 225 μm × 225 μm × 14 (∼20*0.68) μm (thickness).

### mRNA expression analysis

Twenty-six and 30 rats, respectively, were used to analyse the mRNA expression of paxillin and sclerostin (*n* = 8 in C group, *n* = 8 in A25 group, *n* = 10 in A35 group for paxillin mRNA; *n* = 9 in C group, *n* = 11 in A25 group, *n* = 10 in A35 group for sclerostin mRNA). For each rat, a 1 mm slice of bone was cut from a tibia conserved at −80°C in RNA later (Qiagen) with a diamond saw (Dremmel, USA). The sample was placed in a safelock tube (Eppendorf, Hamburg, Germany) with a 5 mm tungsten bead (Qiagen) and was thawed in the tissueLyser II for 1 min. at maximum speed (Qiagen). RNA was extracted from the powder obtained, with a Qiagen RnEasy kit according to the instructions of the manufacturer. The quality and concentration of the RNA were assessed with the RNA stdSens Experion chips (Biorad, Hercules, Germany). The RNA was then reverse transcripted with the RT quantitect kit from Qiagen. The cDNA was stored at −20°C before PCR. PCR was performed with Qiagen quantitect sybr geen PCR kit, in a biorad CX96 cycler. The primers used were designed by Qiagen for amplification of rat *GAPDH* and *RPL13A* as reference genes, and sclerostin and paxillin as interest genes. The primers used were the following: Qiagen QuantiTect Primer Assay: sclerostin (*Sost*): RN_Sost_1_SG (QT00418558), paxillin: RN_Pxn_1_SG (QT01597176), *gapdh*: Rn_Gapd_1_SG (QT00199633), *rp113a*: Rn_Rpl13_1_SG (QT00178675). A melting curve analysis was performed at the end of each PCR to verify purity of the amplifications. The delta-CT method was used for calculations of the ratios. For reference genes, the average of the ratios from the two reference genes was used for normalization [22].

We have chosen to evaluate sclerostin mRNA modulation expression because sclerostin presents the interest to be a late osteocyte differentiation marker, which represents mature osteocyte [13].

### Statistics

Numerical variables were expressed as mean ± SD. Statistical analysis was conducted with the Staview 5.0 software. First, we used the Shapiro–Wilks test to assess the normality of the data distribution. If the data normality was confirmed, Fisher's F test was used to evaluate the homogeneity of the group variances. If the distribution was normal, Student's *t*-test was used to evaluate group differences. When the data were not normally distributed, non-parametric tests were used to assess the difference between groups. The Kruskal–Wallis test was applied, and if significant, a *U*-Mann–Whitney test was realized. Then, the Spearman's rho correlation was achieved to assess the correlation between parameters. The critical *P*-value for statistical significance was *P* = *0.05*. The exact *P*-value was quoted unless it was lower than 0.0001. Changes that have been reported in percentages have been calculated with respect to the C group.

## Results

### Low BMD gain with alcohol consumption

**Fig. 1:**
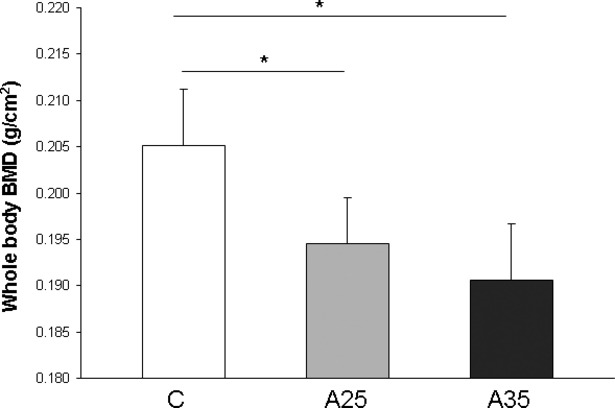
Whole body bone mineral density (BMD) assessed by dual X-ray absorptiometry (Discovery, Hologic, Bedford, MA, USA) at the end of the protocol. Control rats (C group) were compared with rats fed alcohol in the potable tap water for 17 weeks. The alcohol concentration was 25% v/v for A25 group or 35% v/v for A35 group. The whole body BMD was significantly lower in the two alcohol-fed groups compared with the control group. * indicates a significant difference (*P* < 0.05).

As shown in Figure 1, the whole body BMD after the alcohol treatment was significantly (*P* = 0.0002) lower in A35 (0.032 ± 0.006 g/cm^2^) and A25 (0.039 ± 0.003 g/cm^2^) compared with C (0.051 ± 0.005 g/cm^2^). Despite a reduced value, BMD of A35 was not significantly different from that of A25. Nevertheless, this finding clearly indicated that heavy chronic alcohol consumption reduces the BMD accrual in young adult animals.

### Alcohol treatment increases the number of lipid droplets in the bone marrow

**Fig. 2:**
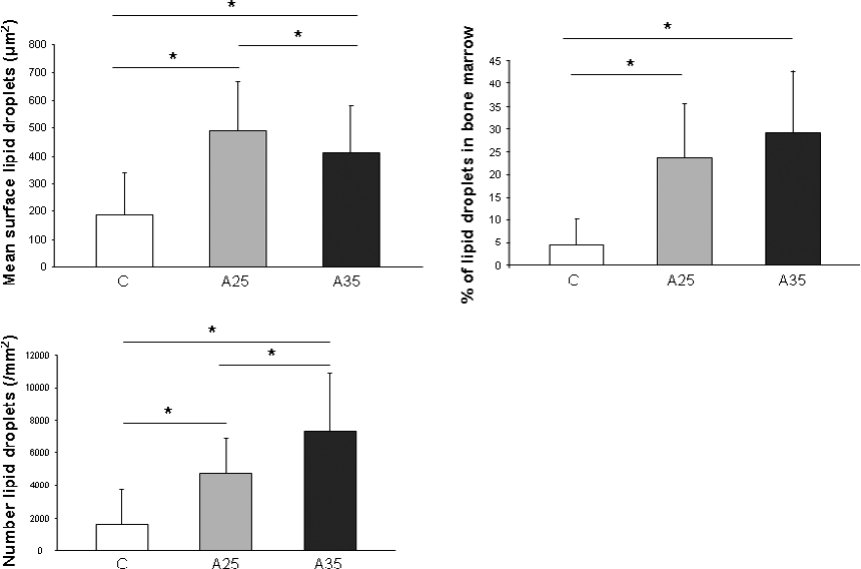
Mean surface, percentage and number of lipid droplets in the bone marrow. Three groups corresponding to seventeen rats were compared (*n* = 4 in C group, *n* = 8 in A25 group, *n* = 5 in A35 group). The percentage of lipid droplets in the bone marrow was significantly higher in A25 and A35 compared with C. There was no significant difference between A25 and A35. The mean surface of lipid droplets was significantly higher in A25 and A35 *versus* C, and higher in A25 compared with A35. The number of lipid droplets was higher in A25 and A35 *versus* C and higher in A35 *versus* A25. * indicates a significant difference (*P* < 0.05).

The adiposity of bone marrow was assessed by Toluidine blue staining to estimate the area occupied by lipid droplets (percentage of lipid droplets), the mean surface of lipid droplet, their number in the bone marrow of the different groups (Fig. 2). The mean surface of lipid droplets was significantly higher (*P* < 0.0001) in alcohol-fed groups (A25: 500 μm^2^; A35: 400 μm^2^) *versus* control rats (C: 200 μm^2^). The mean surface in A25 group was significantly higher (*P* = 0.03) compared with A35. The percentage of lipid droplets in the bone marrow was significantly higher in A25 and A35 compared with C (*P* < 0.0001). However, there was no significant difference between A25 and A35 groups. There were more lipid droplets in the alcohol-fed animals compared with control (A25: 4729.10 ± 2162.95; A35: 7310.37 ± 3534.05 *versus* C: 1619.93 ± 2097.62 with *P* < 0.0001) and the difference observed between A35 and A25 was statistically significant (*P* = 0.005). Overall, these results suggest that chronic heavy alcohol consumption increases the marrow fat content as a result of both increased number and size of lipid droplets.

### Alcohol increases the number of empty osteocyte lacunae

**Fig. 3:**
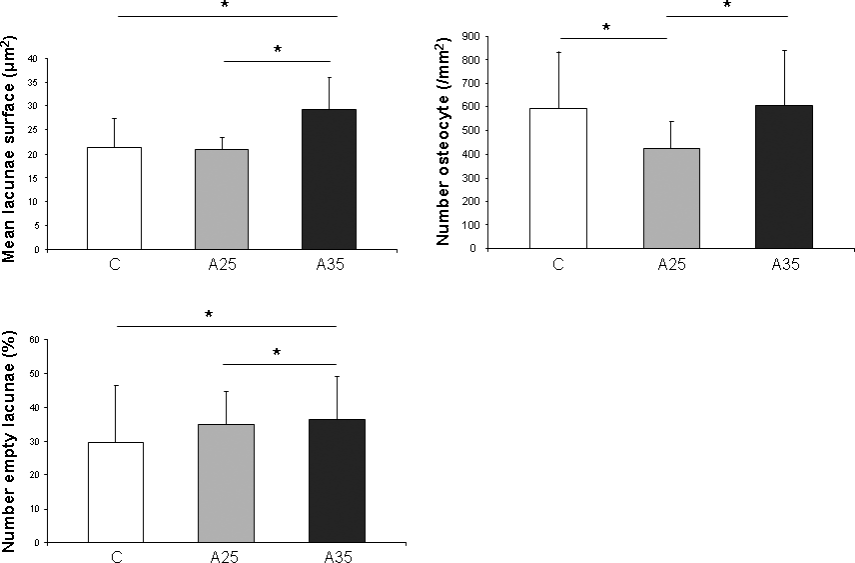
Mean surface of the lacunae, number of empty osteocyte lacunae and number of osteocytes per mm^2^. Nineteen rats (*n* = 6 in C group, *n* = 8 in A25 group, *n* = 5 in A35 group) were used for this quantification. The number of empty osteocyte lacunae was higher in A35 compared with C and A25. The mean lacunae surface was significantly higher in A35 *versus* A25 and C. There was less osteocytes per mm^2^ in A25 *versus* C and A35. * indicates a significant difference (*P* < 0.05).

The effect of alcohol consumption on osteocyte lacunae was also evaluated (Fig. 3). The mean lacunae surface was significantly higher (*P* < 0.0001) in A35 (29.3 ± 6.7 μm^2^) *versus* A25 (20.8 ± 2.6 μm^2^) and C (21.3 ± 6.2 μm^2^). The number of osteocyte/mm^2^ was higher in C and A35 *versus* A25. There was significantly (*P* = 0.0008) more empty osteocyte lacunae in A35 (36.4 ± 12.6%) compared with A25 (34.9 ± 9.9%) and C (29.7 ± 16.6%). These values indicate that heavy alcohol consumption increases the number of empty osteocytes lacunae and the surface of the lacunae.

### The number of osteocytes containing lipid droplets is increased with high alcohol consumption

**Fig. 4:**
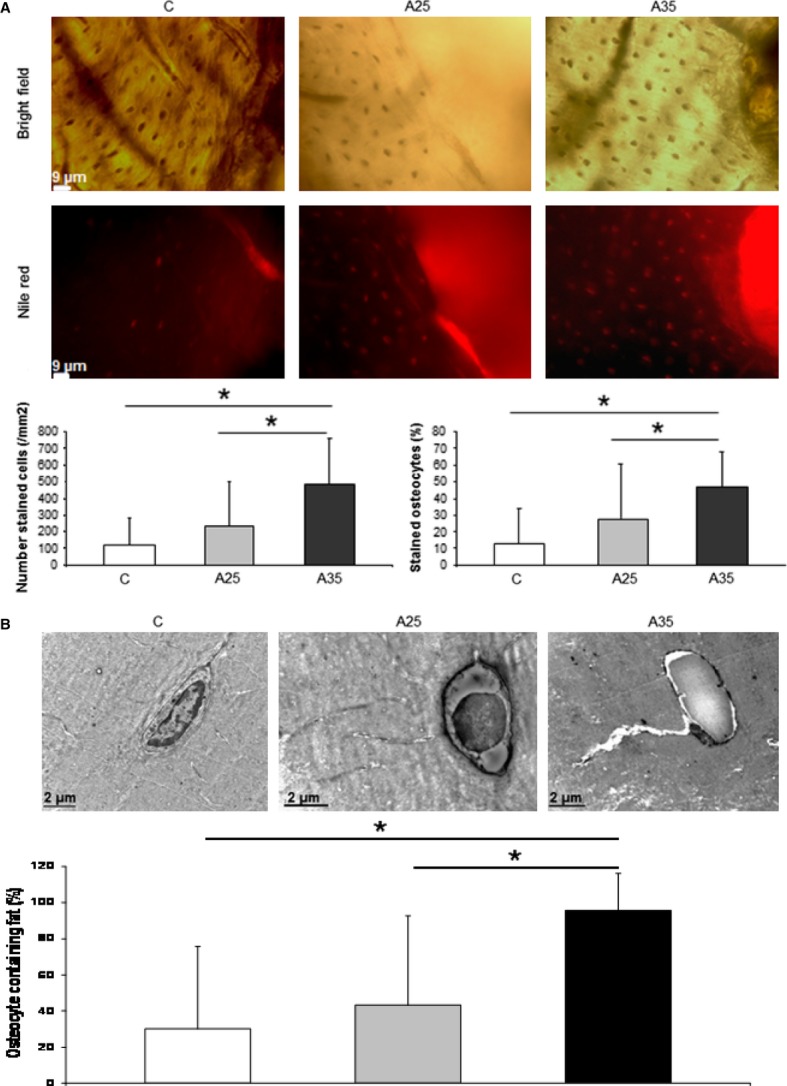
Presence of lipid droplets inside osteocytes has been observed by Nile red staining (**A**) and by transmission electron microscopy (**B**). Tibia slices were stained with Nile red fluorescent hydrophobic dye, based on the published protocol by Fowler and Greenspan [18]. Twenty-three rats were analysed (C group: *n* = 7, A25 group: *n* = 8, A35 group: *n* = 8). Five regions of interest around the cortical bone were chosen and imaged with epifluorescence microscopy (objective magnification 40×). There were more cells containing lipids in the A35 group compared with A25 and C (**A**). The percentage of osteocytes containing lipids (ratio Nile red staining/bright field) was also significantly higher in A35 *versus* A25 and C (**A**). Tibia fragments were dehydrated in alcohols of increasing concentrations and embedded in Epon resin. From the resin blocks, ultra-thin sections (75 nm) were cut and processed as described in Materials and Methods. We observed significantly more osteocytes containing lipid droplets in A35 compared with C and A25 (**B**). * indicates a significant difference (*P* < 0.05).

Nile red staining was performed to estimate the amount of lipid droplets in osteocytes. Nile red dye can specifically detect neutral ubiquitous lipid deposits that are usually not seen with oil red O or other traditional fat stains. We counted significantly more cells containing lipids in the A35 group compared with A25 and C (*P* < 0.0001). The percentage of osteocytes containing lipids (ratio Nile red staining/bright field) was also significantly higher in A35 *versus* A25 and C (*P* < 0.0001) (Fig. 4a), suggesting that the highest alcohol dose is associated with more osteocyte containing fat. Transmission electron microscopy (TEM) analyses allowed us to confirm that there were more osteocytes containing lipid droplets in A35 compared with A25 and C (Fig. 4b).

### Alcohol increases osteocyte apoptosis and the cytoskeleton activity

**Fig. 5:**
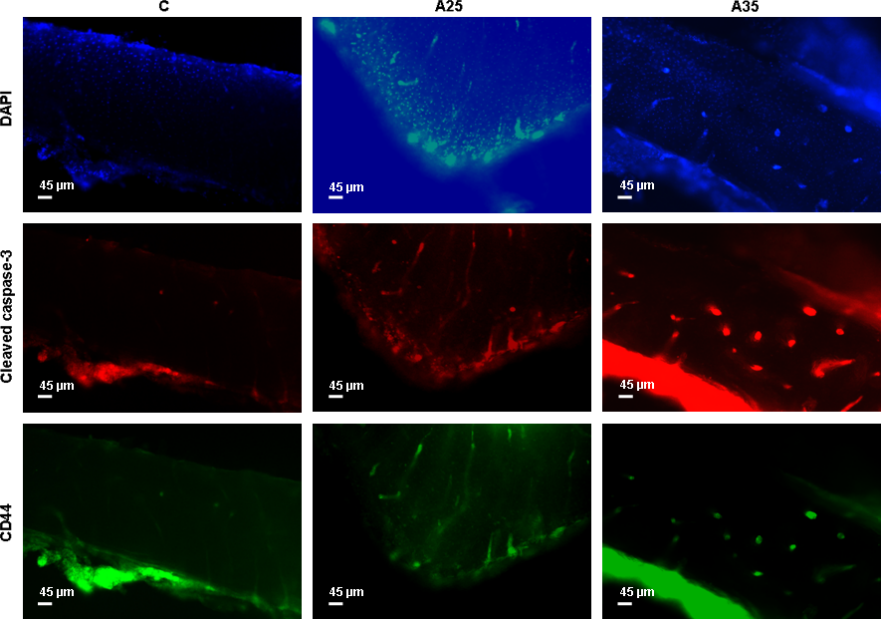
Cleaved caspase-3 and CD44 immunostainings. Bone explants (thickness 400 μm) were cut transversally in the superior part of the tibia diaphysis with a high-speed rotary tool equipped with a diamond saw, without previous embedding. Experiments were conducted with eighteen rats (*n* = 5 in C, *n* = 8 in A25, *n* = 5 in A35). They were immunostained with cleaved caspase-3, which is an early marker of apoptosis, and CD44 as a marker of osteocytes in the mineralized bone. The objective magnification of the epifluorescence images of osteocytes was 10×.

**Fig. 6:**
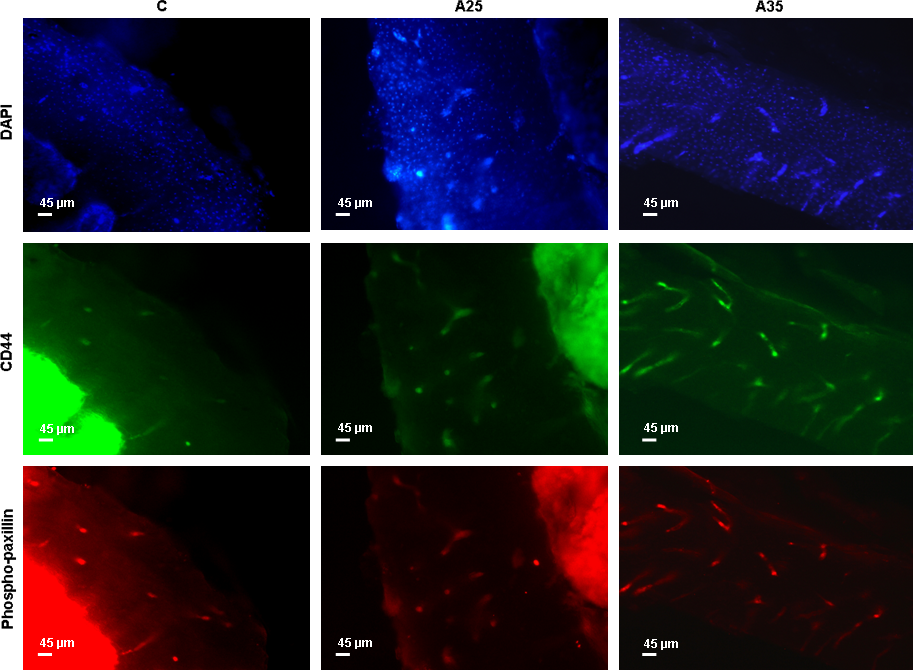
CD44 and phospho-paxillin immunostainings. Bone explants (thickness 400 μm) were cut transversally in the superior part of the tibia diaphysis with a high-speed rotary tool equipped with a diamond saw, without previous embedding. Experiments were conducted with eighteen rats (*n* = 5 in C, *n* = 8 in A25, *n* = 5 in A35). They were immunostained with CD44 as a marker of osteocytes in the mineralized bone, and phospho-paxillin, as described by Vatsa *et al*. [17]. The objective magnification of the epifluorescence images of osteocytes was 10×.

**Fig. 7:**
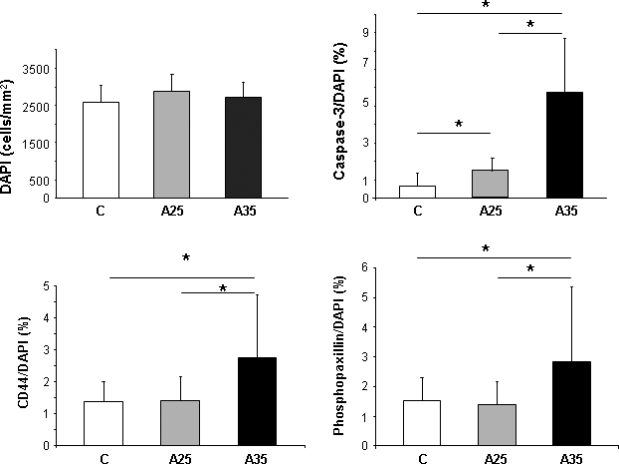
Quantitative results of cleaved caspase-3, CD44 and phospho-paxillin immunostainings. Two series of experiments were performed, one series was achieved with cleaved caspase-3 and CD44, and the second series was achieved with CD44 and phospho-paxillin. Values of CD44/DAPI ratio represent the mean of the both series of experiments. There were significantly more cells stained with cleaved caspase-3 in the A25 and A35 groups compared with the C group. There were also significantly more cells stained with cleaved caspase-3 in A35 *versus* A25. We observed more cells stained with CD44 and phospho-paxillin in A35 group compared with A25 and C, while there was no difference between A25 and C. * indicates a significant difference (*P* < 0.05).

Bone sections were immunostained with cleaved caspase-3, which is an early marker of apoptosis, CD44 as a marker of osteocytes in the mineralized bone, and phospho-paxillin, as described by Vatsa *et al*. (Figs 5 and 6) [17]. There were significantly more cells stained with cleaved caspase-3 in the A25 and A35 groups compared with the C group (*P* < 0.0001) (Figs 5 and 7). There were also significantly more cells stained with cleaved caspase-3 in A35 *versus* A25 (*P* < 0.0001) (Figs 5 and 7). We observed more cells stained with CD44 (*P* < 0.0001) and phospho-paxillin (*P* = 0.0004) in the A35 group compared with A25 and C, while there was no difference between A25 and C (Figs 6 and 7). These data show that alcohol consumption increases osteocyte apoptosis and increases the markers of cell differentiation and cytoskeleton activity.

### Alcohol increases chromatin condensation

**Fig. 8:**
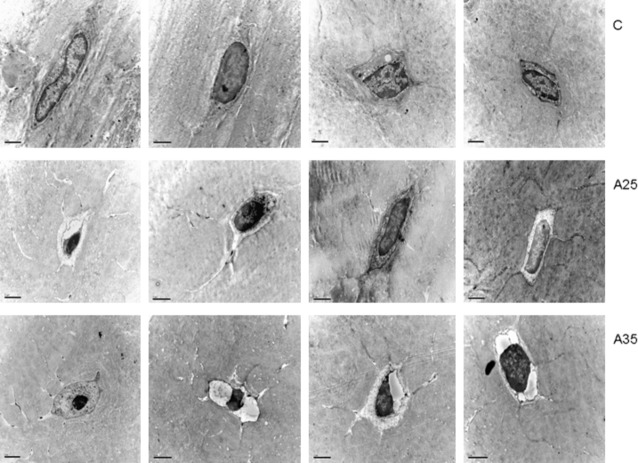
Chromatin aspect of osteocyte nuclei in control (C) rats and in both alcohol-fed (A25 and A35) rats, observed by transmission electron microscopy. Four osteocytes were presented for each group. Black scale bar represents 2 μm.

**Fig. 9:**
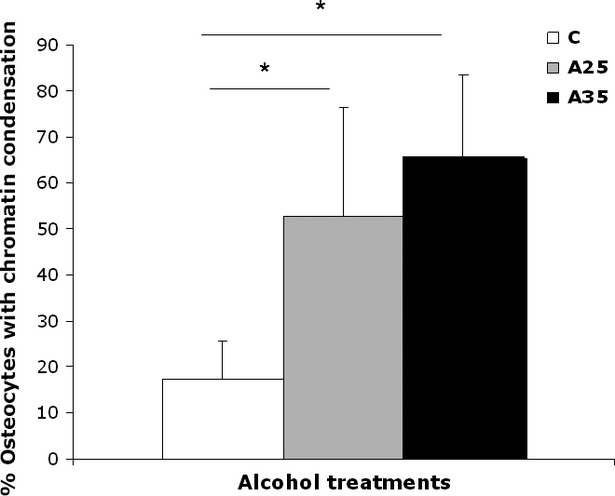
Quantitative results of chromatin condensation of osteocyte nuclei in control (C) rats and in both alcohol-fed (A25 and A35) rats, observed by transmission electron microscopy. Nineteen rats were analysed (C group: *n* = 6, A25 group: *n* = 8, A35 group: *n* = 5). We observed a higher percentage of osteocytes with chromatin condensation in TEM in the A35 group *versus* the C group (65.7 ± 17.8 *versus* 17.3 ± 8.1, *P* = 0.004) and in the A25 group *versus* the C group (52.6 ± 23.8 *versus* 17.3 ± 8.1, *P* = 0.014). * indicates a significant difference (*P* < 0.05).

We observed a higher percentage of osteocytes with chromatin condensation in TEM in the A35 group *versus* the C group (65.7 ± 17.8 *versus* 17.3 ± 8.1, *P* = 0.004) and in the A25 group *versus* the C group (52.6 ± 23.8 *versus* 17.3 ± 8.1, *P* = 0.014; Figs 8 and 9).

### Alcohol is associated with lipid infiltration in osteocytes and dendrites

**Fig. 10:**
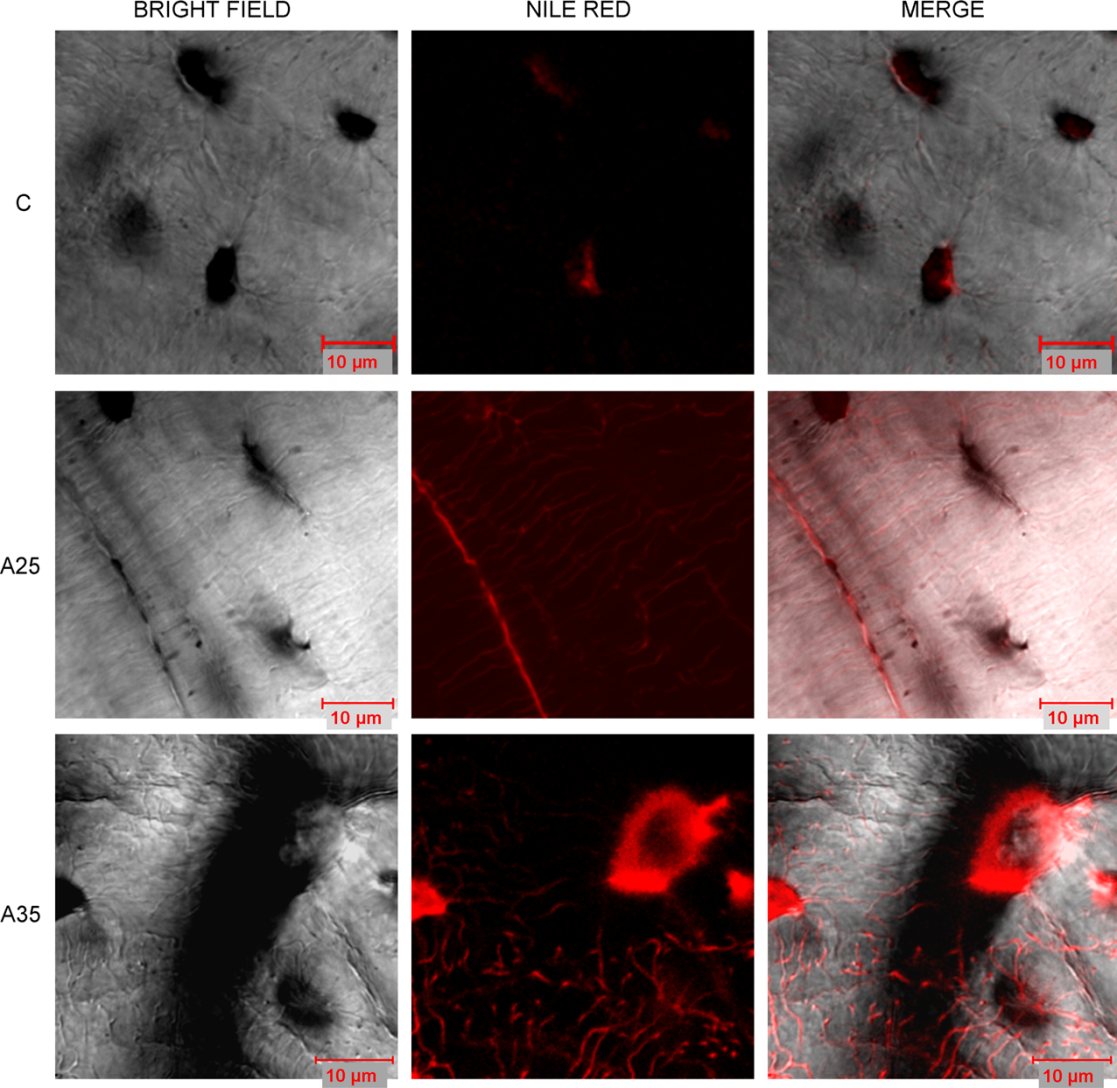
Presence of lipids in the osteocyte network observed by confocal microscopy. Confocal images of osteocytes were obtained on tibia slices fixed in formalin, cut with a high-speed rotatory tool (Dremel) and stained with Nile red as described by Fowler and Greenspan ([18]). A Zeiss Laser Scanning Microscope LSM 510 Meta confocal system attached to a Zeiss inverted microscope (Axiovert 200M; Zeiss) was used (40× oil immersion objective lens). We observed more Nile red staining in the osteocytes of the A35 group compared with those of the A25 and C groups. We observed Nile red staining in the lacuno-canalicular network in the A25 and A35 groups, with more staining in the osteocyte dendrites in the A35 group.

We observed an increase in Nile red staining in the osteocytes of the A35 group compared with those of the A25 and C groups. Nile red staining was found in the lacuno-canalicular network in the A25 and A35 groups, with more staining in osteocyte dendrites in the A35 group (Fig. 10), suggesting that there is a lipid infiltration with alcohol consumption in the osteocyte and dendrites. This lipid infiltration appears to be proportional to the amount of alcohol ingested.

### Paxillin and Sclerostin mRNA expression

**Fig. 11:**
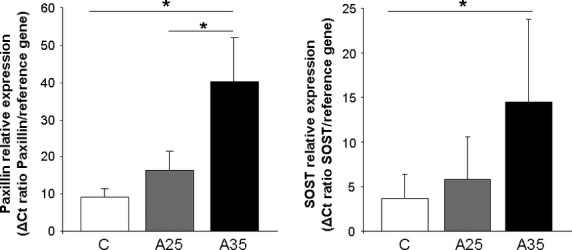
Paxillin and sclerostin mRNA expression in right tibia in control (C) rats and in both alcohol-fed (A25 and A35) rats (*n* = 8 in C group, *n* = 8 in A25 group, *n* = 10 in A35 group for paxillin mRNA and *n* = 9 in C group, *n* = 11 in A25 group, *n* = 10 in A35 group for sclerostin mRNA). Total RNA was extracted from frozen bone tissue and analysed by RT-QPCR as described in materials and methods. Paxillin mRNA expression in the A25 group was not significantly different from control group, but was higher in the A35 group compared with A25 and control groups (A35: 39.2 ± 12.1 *versus* A25: 16.4 ± 5.2, *P* < 0.05; and A35: 39.2 ± 12.1 *versus* C: 9.12 ± 2.23, *P* < 0.05). Sclerostin mRNA expression was higher in the A35 group compared with the control group: A35: 20.8 ± 9.34 *versus* C: 3.23 ± 2.59, *P* < 0.05. * indicates a significant difference (*P* < 0.05).

Paxillin mRNA expression in the A25 group was not significantly different from control group, but was higher in the A35 group compared with A25 and control groups (A35: 39.2 ± 12.1 *versus* A25: 16.4 ± 5.2, *P* < 0.05; and A35: 39.2 ± 12.1 *versus* C: 9.12 ± 2.23, *P* < 0.05; Fig. 11). Sclerostin (*Sost*) mRNA expression was higher in the A35 group compared with the control group: A35: 20.8 ± 9.34 *versus* C: 3.23 ± 2.59, *P* < 0.05 (Fig. 11).

### Correlations between different parameters

We have observed that the percentage of lipid droplets in the bone marrow was inversely correlated with the whole body BMD (*r* = −0.69; *P* = 0.01) and correlated with both cleaved caspase-3/DAPI and CD44/DAPI ratios (respectively, *r* = 0.71; *P* = 0.01 and *r* = 0.57; *P* = 0.04).

We have also noticed that the cleaved caspase-3/DAPI ratio was substantially inversely correlated with the femoral BMD (*r* = −0.76; *P* = 0.002) and with the cortical thickness (*r* = −0.80; *P* = 0.001). The cleaved caspase-3/DAPI ratio was also moderately correlated with the percentage of osteocyte stained with nile red (*r* = 0.62; *P* = 0.01).

A moderate correlation between CD44/DAPI ratio and cleaved caspase-3/DAPI ratio (*r* = 0.55; *P* = 0.02) was also found whilst a substantial correlation was observed between CD44/DAPI and phosphopaxillin/DAPI ratios (*r* = 0.93; *P* = 0.0001). We found a moderate correlation between CD44/DAPI ratio and the percentage of lipid droplets in the bone marrow (*r* = 0.57; *P* = 0.04) and also a moderate inverse correlation between CD44/DAPI ratio and cortical thickness (*r* = −0.62; *P* = 0.01).

We have also observed a moderate correlation between the cleaved caspase-3/DAPI ratio and the percentage of osteocyte with a chromatin condensation (*r* = 0.52, *P* = 0.04).

## Discussion

In this study, we reported that chronic alcohol consumption significantly decreases the whole body BMD according to control conditions. This change is accompanied by an increase in osteocyte apoptosis, an increase in the osteocytes percentage containing lipid droplets, changes in cytoskeleton activity and adhesion proteins, and increase in paxillin mRNA expression for the A35 group *versus* A25.

In the present study, we observed more empty osteocyte lacunae in A35 *versus* C and A25. This may be explained by the higher loss of cells by apoptosis in this group compared with the control group, as observed with cleaved caspase-3 staining and chromatin condensation (Figs 5 and 8). The higher cleaved caspase-3 staining in the A35 group is not accompanied by lower osteocyte number in this group. A decrease in the CD44 expression has been previously associated with an increase in apoptosis in other tissues [23–25]. However, CD44 has also been reported as an anti-apoptotic agent [26–28]. Therefore, an up-regulation of CD44 could happen following heavy alcohol consumption to counterbalance the high apoptosis in the A35 group, and this up-regulation may also show an increase in the differentiation of osteoblasts to osteocytes, to replace the apoptotic osteocytes.

Furthermore, a recent publication has highlighted the joint association of osteocyte apoptosis and differentiation stimulation after thiazolidinedione treatment [29]. In this case, the osteocytic differentiation marker stimulated was sclerostin. Sclerostin presents the interest to be an osteocyte differentiation marker, which represents mature osteocyte [13].

In our study, we have observed that the *Sost* mRNA was 6.42-fold more expressed in the A35 group than in the C group.

We have also observed more osteocytes labelled by CD44 in the A35 group compared with the A25 and C groups. CD44 is clearly known to be an osteocyte differentiation marker [30,31]. We can venture the hypothesis that alcohol could increase the speed of the bone cell turnover, as shown by the increase in osteocyte apoptosis and differentiation (by CD44 and *Sost* mRNA expression) at the same time.

We have observed significantly more osteocytes containing lipid droplets in the A35 group compared with A25 and C groups by TEM, which has been confirmed by Nile red staining. The Nile red staining was moderately correlated with the caspase-3/DAPI ratio, which shows that the infiltration of lipid deposit in osteocyte could be linked to this cell apoptosis. Infiltration of lipids in cells other than adipocytes, is already known to lead to apoptosis in pancreatic β cells [32], cardiomyocytes [33], and hepatocytes [34].

We have found more lipid droplets in the bone marrow of the two alcohol-fed groups compared with the control group. The number of lipid droplets was higher in A35 *versus* A25, while the mean surface of the lipid droplets was higher in A25 *versus* A35. The bone marrow is the site where the mesenchymal stem cells (MSCs) differentiate into specific lineage. The same MSCs can become adipocytes or osteoblasts among other cells [35]. Interestingly, it has been reported that chronic high alcohol consumption changes the balance between adipogenesis and osteogenesis and increases adipogenesis *in vitro* [36,37]. This could explain our observations of higher lipid droplets in the bone marrow in the A25 and A35 groups.

Paxillin is a component of the focal adhesion complexes and is widely used as a parameter to study the distribution of focal adhesions [17]. The interactions between paxillin and α4 integrin subunit are thought to play an important role in the regulation of cytoskeletal remodelling and signalling events that are necessary for cell migration, activation and development [38]. A decrease in paxillin protein expression level might be responsible for the loss of focal adhesion function [39] and a change in paxillin protein redistribution has been observed at the same time as morphological changes in ACHN cells [40].

In our study, we observed a significant increase in cleaved caspase-3 in A35 and A25 *versus* C, while it was significantly higher in A35 *versus* A25. This change in cleaved caspase-3 staining was combined with higher staining for CD44 and phosphopaxillin in A35 only, compared with A25 and C (Figs 5 and 6). Higher CD44 staining in the present study is not surprising as we reported above that CD44 has an anti-apoptotic effect. However, paxillin has also been reported as anti-apoptotic [41]. Paxillin plays a critical role in cell survival signalling [41]. The cleavage of paxillin by caspases might be an important event for focal adhesion disassembly during cell apoptosis, contributing to detachment, rounding and death [41]. Chay *et al*., have clearly observed a decrease in paxillin after an *in vitro* incubation of immunoprecipitated paxillin with a purified active form of caspase-3 in Ba/F3 and COS-7 cells. This decrease was reversed by caspase-3 specific inhibitor [41]. Chay *et al*., have also shown that overexpressed paxillin can inhibit apoptosis [41]. This caspase-mediated cleavage of paxillin could constitute a positive feedback mechanism. In this regulating process, paxillin could be involved in integrin-mediated cell survival.

Increase in paxillin and vinculin proteins expression has also been previously observed at the same time as loss of focal adhesion occurs [42]. The cells may try to compensate for loss of cell adhesion by up-regulating focal adhesion proteins [42]. This phenomenon may also happen in our A35 group, explaining why we observed up-regulation of CD44 and phospho-paxillin proteins but also up-regulation of paxillin mRNA. In a previous paper, we have already shown that high alcohol consumption leads to a change in osteocyte morphology compared with controls, as observed by confocal microscopy [14]. Takenouchi *et al*., have observed that a bacterial toxin induced a redistribution of a number of proteins during morphological ACHN cell changes, among which CD44 and paxillin [40]. During this protein redistribution, Takenouchi *et al*., also noticed transient phosphorylation of paxillin. This is consistent with our observations that high alcohol dose changes osteocyte morphology, which is accompanied by changes in CD44 and phosphopaxillin expression.

We found a substantial correlation between phosphopaxillin/DAPI and CD44/DAPI ratios (*P* = 0.0001; *r* = 0.93). CD44 is both an osteocytic differentiation marker and a transmembrane glycoprotein, which is an integrin-associated molecule [43]. CD44 links extracellular matrix proteins and cytoskeleton [44] and could participate in the rearranging of the focal adhesion complexes induced by alcohol treatment.

To reinforce this, both expressions of phosphopaxillin and CD44 decrease when the cell adhesivity decreases in association with proceeding apoptosis [39]. The cell cytoskeleton is probably disorganized by the high alcohol treatment, as there is a huge lipid droplet infiltration in the cytoplasm as shown on the TEM and confocal images (Figs 4 and 10). We have found lipids in the osteocyte dendrites in A25 and A35 groups and lipid accumulation in osteocyte cell bodies of the A35 group. One possible hypothesis of this phenomenon is that the lipid infiltration in the osteocyte induces a shape deformation in the cell, which modulates the cell adhesion and leads to apoptosis. The correlation observed between cleaved caspase-3 staining and Nile red staining strengthens this hypothesis.

This investigation has a limitation: Unfortunately, we did not successfully amplify *Cd44* mRNA with RTQPCR on mRNA samples directly extracted from bone tissue. Prideaux *et al*., [30] have observed expression of mRNAs pattern of the different osteocytic differentiation markers after cell culture of osteoblasts and osteocytes. They have shown that *Cd44*, among the other osteocytic differentiation markers, is the less expressed in a MLO-A5 cell line on the whole of the time course culture. Furthermore, we worked on bone explants directly, not on cell culture *in vitro*. As a consequence, it is more difficult to obtain mRNAs of targets weakly expressed using this methodology.

In conclusion, this work clearly shows that there is a dose effect of alcohol consumption on cleaved caspase-3 osteocyte immunostaining levels and on the number of lipid droplets in the bone marrow. Between respectively A25 and A35 groups, we have observed an increase in osteocyte apoptosis, an increase in the osteocyte percentage containing lipid droplets, an increase in both paxillin mRNA expression and phosphopaxillin and CD44 proteins levels.

We can hypothesize that the osteocyte apoptosis induced by alcohol, promotes a positive feedback mechanism, which involves CD44 and paxillin. This mechanism could present anti-apoptotic effects and, at a secondary level, contribute to the progression of the osteocytic lineage differentiation.
